# Synchronisation of *Arabidopsis* flowering time and whole-plant senescence in seasonal environments

**DOI:** 10.1038/s41598-018-28580-x

**Published:** 2018-07-06

**Authors:** Matin Miryeganeh, Masaki Yamaguchi, Hiroshi Kudoh

**Affiliations:** 1 Center for Ecological Research, Kyoto University, Hirano 2-509-3, Otsu, 520-2113 Japan; 2 Present Address: Okinawa Institute of Science and Technology Graduate University, Tancha 1919-1, Onna-son, Okinawa 904-0412 Japan

## Abstract

Synchronisation of flowering phenology has often been observed between individuals within plant species. We expected that a critical role of flowering-time control under natural conditions is a phenological synchronisation. However, no studies have quantified the level of synchronisation of reproductive timing relative to germination timing under natural conditions. In a sequential seeding experiment (SSE) in which we manipulated the germination timing of *Arabidopsis thaliana* accessions, we developed a quantification index to evaluate reproductive synchrony in annual plants. In the SSE, we identified a novel phenomenon of reproductive synchrony: senescence synchrony. The role of vernalisation in realising flowering synchrony between plants of different ages under natural conditions was demonstrated by synchronisation and de-synchronisation of flowering initiation in vernalisation-sensitive and less-vernalisation-sensitive accessions, respectively. We also observed up-regulation of senescence-related genes at corresponding times. The approach we developed in this study provides a set of concepts and procedures that can be used to study reproductive synchrony experimentally under natural conditions.

## Introduction

Reproductive synchrony, which is the synchronisation of the reproductive timing between individuals, is essential for successful mating in most organisms^1,2^. Synchronising any developmental event among individuals requires a specific mechanism(s) because individuals differ in age, size, and physiological status. For instance, we often observe synchronisation of biological events, such as phenological synchronisation, among individuals within a range of certain calendar dates^3^. In plants, studies of flowering phenology have identified high levels of reproductive synchrony in diverse plant species^4^.

Flowering time has been considered the most strictly controlled process in plants, and extensive studies have revealed mechanisms that control flowering time^5,6^. In *Arabidopsis thaliana*, nearly 300 flowering-time genes have been identified, and these genes comprise genetic pathways that respond to specific seasonal signals, such as the temperature and photoperiod^7–9^. In previous studies, the effects of particular mutations or environmental stimuli have been primarily evaluated by examining whether they accelerate or delay floral initiation developmentally under laboratory growth conditions. In these studies, flowering-time has primarily been measured as the number of days after germination. However, the hypothesis that plants utilise these mechanisms to synchronise flowering phenology in natural environments has not been tested.

In annual plants, such as *Arabidopsis thaliana*, populations often consist of individuals that were germinated at different times^10^. Earlier experimental studies under natural conditions using *A*. *thaliana* clearly showed that germination timing altered life history characteristics, including the bolting time and flowering time^11,12^. Although these studies manipulated the germination timing and measured the phenotypic plasticity of bolting and flowering dates under natural conditions, they aimed to reveal how germination timing alters life history characteristics and, consequently, influences plant fitness and natural selection. In another line of studies, photothermal models to predict the phenology of *A*. *thaliana* by incorporating the genetic mechanisms of the photoperiod and vernalisation pathways were developed^13^. The models predicted that there is a transition point of germination timing in autumn that causes *Arabidopsis* accessions to switch their flowering time between “before” and “after” winter, and later germination was predicted to result in longer days to bolting^13^. Based on these experimental and modelling studies, we suspected that the critical role of flowering-time control under natural conditions is phenological synchronisation between individuals that germinate at different times. However, no studies have quantified the level of synchronisation of reproductive timing relative to germination timing.

Although the mechanisms controlling the bolting and flowering time have been studied extensively, how the phenology of reproductive termination and whole-plant senescence are controlled have been less explored^14^. Before the end of reproduction in annual plants, active translocation of resources from leaves to reproductive organs should take place at the expense of successive leaf senescence^15^. Breeding against the onset of senescence in crops has been considered to be a strategy to increase crop productivity^16^, which suggests that the timing of senescence can be another target of natural selection^14^. The length of the period between bolting and whole-plant senescence has been shown to be highly heritable in green-house experiments using 45 accessions of *A*. *thaliana* and recombinant inbred lines between Ler-0 and Cvi-0^17^. Therefore, whether senescence timing is synchronously controlled in *A*. *thaliana* is a novel question that should be addressed.

**Figure 1:**
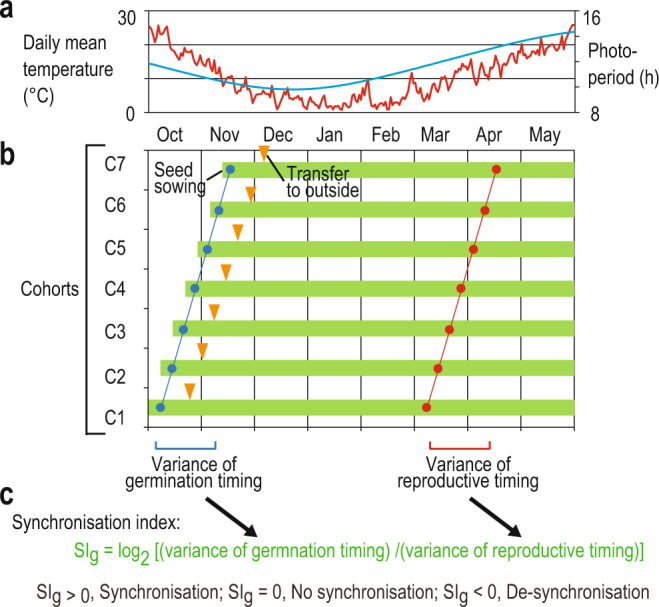
Design of the sequential seeding experiment (SSE) and synchronisation index (SI_g_). (**a**) Daily mean temperatures (red) and photoperiods (blue) during the experiment, (**b**) design of the SSE, and (**c**) formula for calculation and interpretation of the SI_g_ values. In SSE, seven cohorts (C1**–**C7) were prepared by sowing seeds at one-week intervals from the beginning of October to early December. Triangles indicate transfer to the outside garden at 21 days after seed sowing (**a**).

We conducted a sequential seeding experiment (SSE) in which we measured the degree of phenological synchronisation of flowering and senescence against variations of germination timing (Fig. 1a,b). In the SSE, we prepared seven groups of plants, each of the same age (a cohort), by seeding seven times at one-week intervals (hereafter referred to as C1–C7; Fig. 1b). When we compared the seven cohorts on a given calendar date, each cohort was seven days older than the next one. By quantifying variation of the reproductive phenology between cohorts relative to that of germination timing, we quantified the degree of phenological synchronisation of reproductive events, such as bolting, flowering and senescence. We developed a novel synchronisation index to evaluate phenological synchronisation against variation in germination timing (Fig. 1c), i.e.,

1 $${{\rm{SI}}}_{{\rm{g}}}={\mathrm{log}}_{{\rm{2}}}[({\rm{variance}}\,{\rm{of}}\,{\rm{germination}}\,{\rm{timing}})/({\rm{variance}}\,{\rm{in}}\,{\rm{timing}}\,{\rm{of}}\,{\rm{the}}\,{\rm{event}}\,{\rm{of}}\,{\rm{interest}})]$$

Positive SI_g_ values represent synchronisation (e.g., SI_g_ = 1 and 2 correspond to halved and quartered variance, respectively), while negative values represent de-synchronisation (e.g., SI_g_, = −1 and −2 correspond to doubled and quadrupled variance, respectively). We expected to observe phenological synchrony (SI_g_ > 0) if plants had season-dependent regulation, a specific mechanism that shifts the timing of a reproductive event towards a certain calendar date. We further examined the time-series changes of expression in selected flowering-time and senescence-related genes.

Specifically, we asked the following questions: (1) Do plants with different germination timing indicate synchrony in the timing of flowering and senescence under outside seasonal conditions (does a positive SI_g_ exist)? (2) Do the pattern and degree of synchronisation differ between flowering and senescence timing or between accessions with large differences in their vernalisation requirements? (3) Could we observe upregulation of senescence-related genes at the time of senescence synchrony?

## Results

### Flowering initiation is synchronised in Lov-5 and Tamm-2 but de-synchronised in C24 and Ler-1

**Figure 2:**
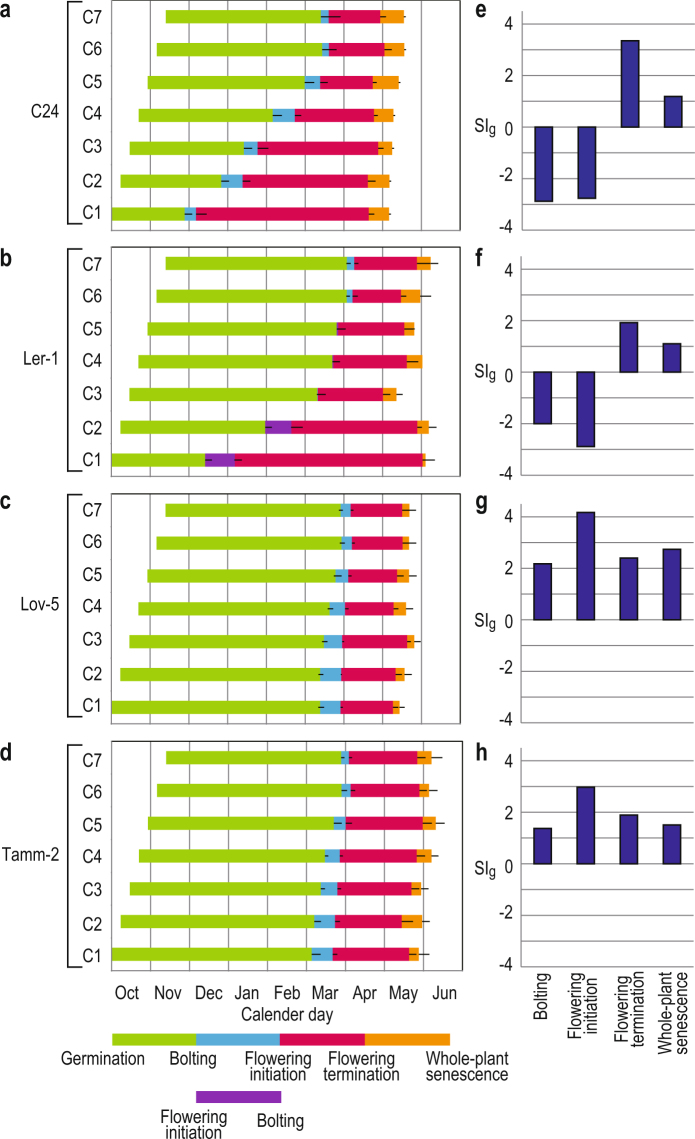
Results of SSE for four accessions of *Arabidopsis thaliana*. Phenological responses of the seven cohorts for (**a**) C24, (**b**) Ler-1, (**c**) Lov-5, and (**d**) Tamm-2 are indicated by bars representing the periods between germination and four successive reproductive timings. The SI_g_ values of bolting, flowering initiation, flowering termination, and whole-plant senescence are presented for (**e**) C24, (**f**) Ler-1, (**g**) Lov-5, and (**h**) Tamm-2. In (**a–d**), the colours correspond with those in the bars at the bottom of the figure. In C1 and C2 of Ler-1, flowering initiation took place before bolting.

We conducted a series of SSE using four accessions of *Arabidopsis thaliana* (i.e., C24, Ler-1, Lov-5, and Tamm-2). The former two and latter two were known as early- and late-flowering accessions, respectively. Lov-5 and Tamm-2 require strong vernalisation (i.e., exposure to prolonged cold) before flowering, but C24 and Ler-1 flower relatively early, even without vernalisation^18-20^. In the SSE, we measured the timing of bolting, flowering initiation (opening of the first flower), flowering termination (arrest in the production of new flowers), and whole-plant senescence of the seven cohorts (Fig. 2a–d). Strong de-synchronisation (SI_g_ < -2) and synchronisation (SI_g_ > 1) were detected for early- and late-flowering accessions for bolting and flowering initiation, respectively (Fig. 2e–h). The SI_g_ values for bolting and flowering initiation indicated that variance across the seven cohorts became 4–8 times larger than that for the germination timing in C24 and Ler-1, whereas the variation was 1/2-1/16 for Lov-5 and Tamm-2 (Fig. 2e–h). Regarding C24 and Ler-1, early-germinated cohorts flowered before winter, but late-germinated cohorts flowered in spring, and these responses resulted in de-synchronisation of bolting and flowering initiation in early-flowering accessions (Fig. 2a,b). Regarding Lov-5 and Tamm-2, bolting and flowering initiation occurred synchronously in spring for all cohorts (Fig. 2c,d). The timing of bolting and flowering initiation in these accessions with strong vernalisation requirements was regulated in a seasonally dependent manner.

### Senescence is synchronised in all accessions

All accessions indicated strong synchronisation of senescence-related events, such as flowering termination and whole-plant senescence (positive SI_g_; Fig. 2e–h). The SI_g_ values for flowering termination and whole-plant senescence indicated that variances across the cohorts became approximately 1/10, 1/4, 1/5 and 1/4, and 1/2, 1/2, 1/7 and 1/3 of the variance of germination timing for C24, Ler-1, Lov-5 and Tamm-2, respectively (Fig. 2e–h). Synchronisation of these traits was observed in both early- and late-flowering accessions, and de-synchronisation in flowering initiation in the former group did not affect the synchronisation of senescence-related traits. Overall, season-dependent regulation of timing was suggested for flowering termination and whole-plant senescence in all accessions.

### C24 and Ler-1 flower after constant PTU

**Figure 3:**
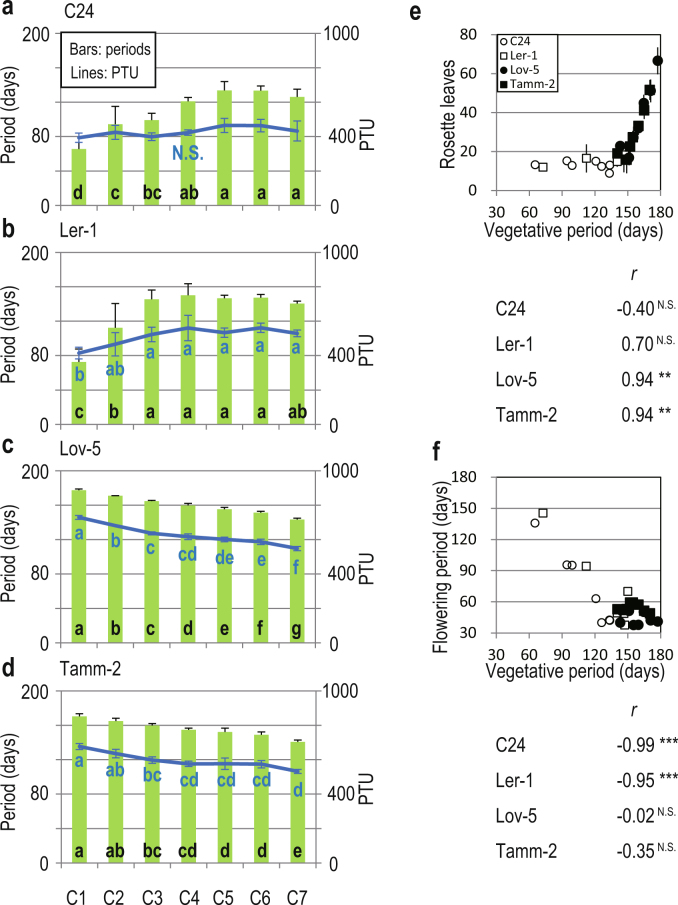
Comparison of the vegetative periods and PTU across cohorts in the four accessions and the leaf number at bolting and flowering period compared to the vegetative period. Duration of vegetative periods (green bars) and PTU values during the corresponding periods (lines) are presented for seven cohorts of (**a**) C24, (**b**) Ler-1, (**c**) Lov-5, and (**d**) Tamm-2. (**e**) The dependency of the rosette leaf number at bolting on the vegetative period, and (**f**) the relationship between vegetative and flowering periods. The duration of the vegetative periods was calculated as the number of days from germination to flower initiation. In (**a**–**d**), the means and standard deviations (SD) are presented. Different letters at the bottom of the bars and next to the lines indicate significant differences (*P* < 0.05) in periods and PTU values between cohorts. N. S. represents no significant difference detected between all combinations of cohorts. In (**e**,**f**), the cohort means are plotted with different symbols for four accessions. Correlation coefficients (*r*) are also listed for each accessions (^***^, ^**^, N.S.; *P* < 0.001, *P* < 0.01, no significance at *P* < 0.05, respectively). Standard deviations for the number of rosette leaves are represented by vertical bars (**e**).

The SSE was set at the beginning of October, and later cohorts started growth on days with smaller photothermal units (PTU) that resulted from lower temperature regimes and shorter photoperiods (Fig. 1a,b). PTU is a scale of temperature for growth perceived by plants and is calculated using temperature and photoperiod records. PTU-dependent regulation of flowering-time (flowering after crossing a certain threshold of accumulated PTU) has been incorporated in the phenology model of *A*. *thaliana*^13^. To examine whether de-synchronisation of bolting and flowering initiation for C24 and Ler-1 are explained by PTU-dependent regulation, we calculated the vegetative PTU (PTU from the time of germination to flowering initiation) for all cohorts of four accessions (Fig. 3a–d). For C24 and Ler-1, constant vegetative PTU values were observed across the seven cohorts, except for a smaller PTU in C1 of Ler-1 (Fig. 3a,b). The number of rosette leaves at bolting was coincidently constant across cohorts in C24 and Ler-1 (Fig. 3e). This result suggested that earlier cohorts of these accessions flowered even before winter after producing a constant number of leaves when they perceived a constant amount of PTU.

The vegetative periods of Lov-5 and Tamm-2 became shorter from C1 to C7 at almost constant intervals (5.7 and 4.9 days, on average, between successive cohorts, respectively), and longer vegetative periods resulted in higher vegetative PTU values (Fig. 3c,d). Longer vegetative periods resulted in higher numbers of rosette leaves (*r* = 0.94 for both Lov-5 and Tamm-2, Fig. 3e). Flowering periods were shorter in later cohorts in C24 and Ler-1 and were almost constant in Lov-5 and Tamm-2 (Supplementary Fig. S1a–d) because flowering termination was strongly synchronised for all accessions. This incidentally resulted in a negative relationship between the vegetative and flowering periods in C24 and Ler-1 (*r* = −0.99 and −0.95, respectively, Fig. 3f). The rosette size at bolting and fruit production could not be simply explained by either the vegetative/flowering period or PTU (Supplementary Fig. S1e–j). We initially expected that a plant that had more PTU would become larger and make more fruits, but some previous studies have pointed out that the meristem limitation (reduction of growth and fruit production due to loss of meristem activity after longer vegetative period) could obscure the expected pattern^21,22^.

### Senescence-related genes are upregulated before flowering termination

**Figure 4:**
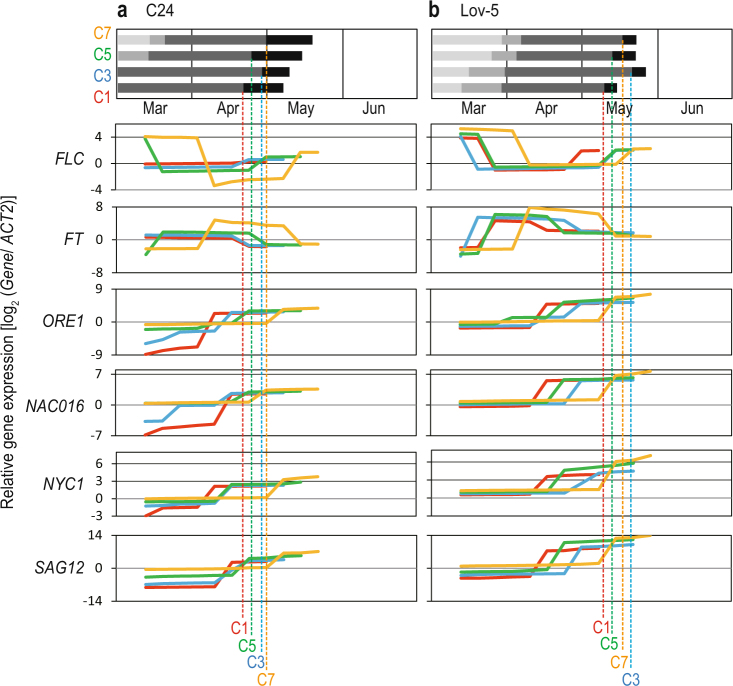
Time-series changes in gene expression of two flowering-time genes (*FLC* and *FT*) and four senescence-related genes (*ORE1*, *NAC016*, *NYC1*, and *SAG12*) before flowering termination. Patterns in C1, C3, C5, and C7 are indicated by thick red, blue, green, and orange lines, respectively, for (**a**) C24 and (**b**) Lov-5. Gene expression (log_2_ relative to those of *ACT2*) of the least-senesced leaves of plants was measured weekly from early March. The median values of two replicates at each sampling day are indicated. The average timings of flowering termination are represented by vertical dashed lines with corresponding colours. The top diagrams represent the timing of bolting, flowering initiation, flowering termination, and whole-plant senescence (boundaries between light-grey – grey – dark-grey – black, and end of the black bars, respectively) during the period of March–June in the SSE. The results including all examined genes and accessions are listed in Supplementary Fig. S2.

We examined the expression pattern of representative flowering-time and senescence-related genes using samples from C1, C3, C5, and C7 to determine whether expression was altered before flowering termination (Fig. 4). One or two leaves from each cohort of every accession were harvested at noon every week beginning on 12 March 2014. We selected leaves for sampling among the fully-expanded youngest leaves available. This procedure resulted in sampling the least-senesced leaves from individual plants. For flowering-time genes, we examined the expression of *FLOWERING LOCUS C* (*FLC*) and *FLOWERING LOCUS T* (*FT*). *FLC* is a suppressor of flowering^23^, and *FT* encodes a florigen, which is a mobile flower-promoting signal^24^. We observed *FLC* suppression and *FT* upregulation during flowering periods both in early- and late-flowering accessions (Fig. 4, Supplementary Fig. S2). *FT* expression is known to show circadian oscillations^24^, and we sampled at noon throughout the experiment. A slight rise in *FLC* expression was observed at the end of flowering before whole-plant senescence (Fig. 4). This observation may be related to the previous finding that DNA replication is required for the maintenance of vernalisation-induced repression of *FLC*^25^, but further investigations are required to address the cause of the observed *FLC* upregulation.

We chose seven representative genes that positively regulate leaf-senescence: *ORESARA 1* (*ORE1*), *NAC-LIKE ACTIVATED BY AP3/PI* (*NAP*), *NO APICAL MERISTEM/ARABIDOPSIS TRANSCRIPTION ACTIVATION FACTOR/CUP-SHAPED COTYLEDON 016* (*NAC016*), *STAY-GREEN 1* (*SGR1*), *NON-YELLOW COLORIMG 1* (*NYC1*), *SENESCENCE 4* (*SEN4*), and *SENESCENCE-ASSOCIATED GENES 12* (*SAG12*). Before flowering termination, upregulation of these genes was observed in all cases except for *ORE1*, *NYC1* and *SAG12* in C7 of C24 (Fig. 4), which implied active translocation of resources from leaves to reproductive organs and successive leaf senescence. Upregulation of *ORE1*, *NAC016*, *NYC1*, and *SAG12* of C1, C3, C5 and C7 occurred within periodical ranges of 4 weeks or shorter in both C24 and Lov-5, although the germination timing differed by 6 weeks between C1 and C7 (Fig. 4). Similar patterns were observed for other genes and accessions, and upregulation of senescence-related genes occurred according to the order of either cohort (C1, C3, C5 and C7) or flowering termination but synchronously relative to germination (Supplementary Fig. S2). Both age and season affected the timing of the upregulation of the level of gene expression for the genes we examined in this study.

## Discussion

Senescence synchronisation among plants of different ages was identified under seasonal environments. To the best of our knowledge, this is the first experimental demonstration of senescence synchrony in annual plants. In our experiment, synchronisation of senescence was not a side-effect or by-product of the synchronisation of flowering initiation. The difference in germination timing de-synchronised the timing of bolting and flowering initiation in C24 and Ler-1. Moreover, these accessions also indicated strong synchronisation of flowering termination and whole-plant senescence. Accessions that indicated synchronised bolting and flowering initiation (Lov-5 and Tamm-2) also showed strong synchronisation of senescence. Therefore, it is likely that the timing of flowering termination and whole-plant senescence is under season-dependent regulation, independent of the timing of bolting and flowering initiation. Apparent negative correlations between vegetative and flowering periods across the cohorts were observed in C24 and Ler-1 due to strong senescence synchrony.

It is possible that plants can die simultaneously due to unfavourable external conditions, but this was not the case in our experiment. The daily mean temperature was mostly in the range between 15 °C and 25 °C when senescence occurred (Fig. 1a), and these temperatures are within the range of the normal growth conditions often used in laboratory studies^26^. Furthermore, plants were watered regularly to avoid occasional drought. The synchronised senescence of C24 occurred approximately one month earlier than that of Tamm-2, and therefore, C24 terminated flowering when Tamm-2 was actively producing flowers. Therefore, we deduced that the observed senescence was controlled internally by the plants in response to seasonal environments.

Upregulation of senescence-related genes was indicated in leaves before flowering termination. We sampled the least-senesced leaves of every individual plant, and the gene expression pattern reflected the senescence timing of the last portion of source organs (i.e., leaves) during whole-plant senescence. The seven genes we examined in this study have been reported to be upregulated during leaf senescence. *ORE1*, *NAP* and *NAC016* encode major senescence-promoting *NAC* transcription factors and upregulate the number of senescence-associated genes (SAGs)^27–30^. *SGR1* and *NYC1* are representative SAGs that encode a chlorophyll catabolism-associated protein and a chlorophyll b reductase, respectively^31^. *SEN4* and *SAG12* are other representative SAGs that are widely used as molecular markers of leaf senescence^32,33^. Upregulation of these genes 2–4 weeks before flowering termination is another indication that plants control when to terminate reproduction by actively translocating resources from leaves to reproductive organs at the expense of individual leaf senescence. Although it has been reported that long days accelerate leaf senescence at the whole-plant level in eight accessions of *A*. *thaliana*^34^, further information is required to connect the regulation of leaf senescence genes to changes in plant life history^14^. In future studies, it will be necessary to identify the processes that trigger flowering termination and whole-plant senescence in response to seasonal environmental stimuli to understand the mechanisms behind senescence synchrony.

The functional significance of senescence synchrony is a novel question to be addressed. Once annual plants enter the reproductive phase, there are still two possible paths of action. One is to translocate all resources from vegetative organs into seeds, leading to whole-plant senescence, and the other is to maintain the vegetative parts and allocate excess photosynthates to seed production^35^. The life-history theory predicts that the decision will be made by the balance between current and future reproduction to maximise lifetime fecundity^22,36^. We expect that there are factors that lower seed production during summer in the original habitats of *A*. *thaliana* accessions.

*Arabidopsis thaliana* accessions exhibit a large natural variation of flowering time^37^. Late-flowering behaviour is primarily caused by the interaction of two genes: *FLC* and *FRIGIDA* (*FRI*), which are positive regulators of *FLC* expression^20^. Most early-flowering accessions have low *FLC* expression and/or a non-functional *FRI* gene^38^. Ler-1 has a low *FLC* expression level due to the reduced sensitivity to FRI of the *FLC* allele^39^. The C24 ecotype contains a unique combination of an *FRI* allele that delays flowering and an *FLC* allele that suppresses the late-flowering phenotype of *FRI*^40^. Conversely, Lov-5 and Tamm-2 are characterised by combinations of *FLC* and *FRI* alleles that require longer vernalisation treatments to fully suppress *FLC*^20^. The active *FRI-FLC* regulatory system is considered to be the mechanism that enables flowering after winter. Our SSE demonstrated another function of the *FRI-FLC* regulatory system, the strong synchronisation of flowering time across plants of different ages. The chronological variance of the timing of first flower opening among cohorts was approximately 1/16 and 1/8 of those at germination for Lov-5 and Tamm-2, respectively. This response involved developmental responses that altered the leaf number at bolting.

In early-flowering accessions (C24 and Ler-1) with weak *FLC*-mediated regulation, bolting and flowering initiation were de-synchronised. The variance among cohorts was approximately eight times greater than the variance in germination timing for both accessions at the time of flowering initiation. This de-synchronisation of flowering occurred without altering the leaf number at bolting. It is likely that when the *FRI-FLC* system had a weak influence on flowering time, the timing of flowering was best explained by PTU-dependent regulation. We predicted that *A*. *thaliana* has a basal response in which it flowers after experiencing a certain amount of PTU. Regarding adaptation to the seasonal environments, season-dependent regulation has become more important than PTU-dependent regulation in habitats where fitness loss by flowering before or during winter is serious. The observed de-synchronisation can be interpreted as the switch between ‘winter-annual’ and ‘rapid-cycling’ life cycles that was predicted to be produced by most *Arabidopsis* accessions through phenotypic plasticity according to the model of seasonal life history plasticity^13^. Even earlier cohorts that initiated flowering before winter produced flowers and fruits actively after winter and terminated their reproduction in late spring. The contrast between ‘winter annual’ and ‘rapid-cycling’ cannot be applied simply in terms of senescence timing.

Synchronous reproduction has long been observed in plant ecology^3^. Synchronous reproduction is required for mating to exchange pollen among individuals and for successful seed maturation and optimal seed dispersion under favourable conditions^41^. Conventional indices of reproductive synchronisation quantify the overlap of reproductive periods between individuals. In this study, we presented a novel definition and quantification method to evaluate reproductive synchronisation by connecting phenological synchronisation with life-history plasticity. The roles of an environmental regulatory system in synchronisation or de-synchronisation of phenological events can be tested by performing SSE experiments and using the synchronisation index developed here. This approach is a strong methodology for molecular phenology^42^ to further understand how phenological synchronisation is regulated at the molecular level.

## Methods

### Plant materials and sequential seeding experiments (SSE)

SSE was conducted in a natural environment using an outside experimental garden at the Center for Ecological Research, Kyoto University (34° 58ʹN, 135° 57ʹE, 150 m elevation), which has a temperate climate with moderate seasonal temperatures and photoperiodic changes (Fig. 1a). Seeds of all accessions were obtained from “The *Arabidopsis* Biological Resource Center (ABRC) - the Ohio State University” (http://www.arabidopsis.org/abrc/). The seed sowing dates of C1, C2, C3, C4, C5, C6, and C7 were 4, 11, 18, and 25 October and 1, 8, and 15 November 2013, respectively. Plants were transplanted into pots 21 days after germination and then transferred to the outside experimental garden. Plants were successively grown in the outside seasonal environment until the end of the experiment. Forty to fifty surface-sterilised seeds of each accession were sown on the surface of the culture medium in Petri dishes at each seeding date, and each dish contained 40 ml of Murashige and Skoog media. After the seeds were kept at 4 °C in darkness for three days to break dormancy, they were moved to an incubator (Growth Cabinet- HNM- S11, Koito Industries, LTD., Japan), where they received approximately 400 µmol m^−2^ s^−1^ of photosynthetically active radiation (PAR) during a 12 h photoperiod, with a day/night temperature of 20 °C. After 21 days, seedlings were transplanted into 10 cm diameter clay pots including coarse-grained loamy soil in the lower half of the pot and a 1:1 mixture of fine-grained pumice and peat in the upper half. We added a slow-acting fertiliser (Mag Amp K, HYPONeX, JAPAN; N:P:K:Mg = 6:40:6:15) according to the manufacturer’s instructions (50 g per 12 L of mixed soil). Pots and soils were prepared two days before transplantation of each cohort. We prepared 18 replicates for each cohort of each accession—6 and 12 of replicates were used for phenology observations and RNA sampling, respectively. We prepared 12 plants for RNA-sampling to minimise the damage to the plants because it required repeated sampling of leaves.

The dates of plant transfer to the outside garden were 25 October, 1, 8, 15, 22, and 29 November, and 6 December 2013 for C1, C2, C3, C4, C5, C6, and C7, respectively. Pots were distributed using a randomised block design across twenty-four 1.2 × 1.2 m woody blocks filled with decomposed granite soil. All combinations of accessions were represented in each block, and the positions of the accessions and cohorts within each block were randomised. The soil surface temperature was recorded every 30 min using a HOBO Water Temp Pro v2 temperature logger (Onset Computer Corporation, Bourne, MA, USA) throughout the experiment. The logger was set beneath the surface of the soil in a pot without a plant and placed inside one of the blocks in the common garden. Plants were watered to reduce mortality after transplantation. Approximately 3% of plants died within 1–2 days after transplantation. These plants were replaced with replicate plants from the same accession when possible until two weeks after transplantation. All of these processes were repeated every week for seven weeks to make seven different cohorts. The final numbers of phenology-monitored plants per cohort were six for all accessions except C4 of C24 and C4 of Lov-5 (five plants for each). Plants were watered three times per week unless there was enough rain. We monitored and recorded the time of bolting, flowering initiation and termination, and whole-plant senescence. Bolting was defined as the elongation of main stems longer than 3 mm. Whole-plant senescence was defined as the day that all vegetative and reproductive parts were completely withered. For the calculation of SI_g_, we first calculated the cohort-means of an event and then the variances across cohort-means. The number of leaves and diameter of the rosette at the time of bolting were recorded. The total number of fruits produced by each plant was also recorded.

### PTU during vegetative and flowering periods

We calculated the duration of two specific periods, the vegetative and flowering periods, to represent phenological responses. The former was defined as the period from germination to flowering initiation, and the latter was defined as the period between flowering initiation and termination. Bolting has often been considered to be the first morphological sign of transition from the vegetative to reproductive phase. In our experiments, bolting and flowering initiations occurred closely, and whether we used bolting or flowering initiation as the transition timing between vegetative and flowering periods did not alter the conclusions. The vegetative and flowering periods are also expressed as PTU values. The temperature in the outside garden was obtained every 30 minutes using a temperature logger until the end of the experiment. Photoperiod records were obtained from the website of the National Astronomical Observatory of Japan (NAOJ, http://eco.mtk.nao.ac.jp/koyomi/dni/dni26.html).

PTU (degree days)^13^ was calculated as the cumulative temperature over a basal threshold base temperature (µ_*b*_) during the daytime:

2 $${\rm{PTU}}={\sum }_{i=g\,{\rm{or}}\,fi}^{fi\,{\rm{or}}\,ft}{\rm{\lambda }}({{\rm{\mu }}}_{{\rm{L}}i}-{{\rm{\mu }}}_{b})$$

where *i* spans from the germination date (*g*) to the timing of flowering initiation (*fi*) for the vegetative period or from *fi* to flowering termination (*ft)* for the flowering period. λ_*i*_ is the daily photoperiod as a proportion of 24 h, counting only the days with mean temperatures during daylight hours, µ_L*i*_, greater than µ_*b*_. In this study, we applied µ_*b*_ = 3 °C, which has been reported as the optimised base temperature in modelling the developmental rate of Col-0^43^.

### Statistical analyses

For each accession, differences between cohorts in the vegetative and flowering periods and in vegetative and flowering PTU were tested by Duncan’s multiple comparison tests at *P* < 0.001 using “multcomp” in R software^44^. We also calculated the correlation coefficients (*r*) across 7 cohorts between the vegetative period and number of rosette leaves and between the flowering and vegetative periods for each accession.

### RNA sampling and extraction

Time-series RNA sampling was performed periodically before senescence synchrony for C1, C3, C5, and C7. From each cohort of every accession, leaves were harvested at noon every week starting from the beginning of March 2014. For each cohort and accession, leaves were sampled from two plants separately and treated as two biological replicates (one to two leaves per replicate). We sampled leaves by selecting from the youngest leaves available. This procedure enabled sampling of the least-senesced leaves from individual plants. At each sampling time, we randomly selected two plants that had not been sampled from 12 replicates for each cohort and accession, and plants were used for the second round from the 7th sampling following the same procedure as long as leaves were available. The leaf samples were put in 2 ml microtubes containing 3.2 mm stainless steel beads, immediately preserved in liquid nitrogen and kept at −80 °C until RNA extraction.

Samples were pulverised using a Multi-Beads Shocker (Yasui Kikai, Osaka, Japan) at 2,000 rpm for 60 s. Total RNA was purified from ground tissue samples with a Promega Maxwell 16 kit (Promega, Madison, WI, USA) according to the manufacturer’s instructions, including a DNase treatment step to remove possible genomic DNA contamination. The RNA concentration was determined using a Nanodrop® spectrophotometer ND-1000 (Nanodrop technologies, Wilmington, DE, USA) and a Quantus fluorometer (Promega, USA). The RNA quality was assessed using a Bioanalyser 2100 (Agilent Technologies Canada, Inc., Mississauga, ON, Canada). High-quality RNA with a 260/230 ratio, an absorbance 260/280 ratio greater than 2 and an RNA integrity number (RIN) above 7 was used for cDNA syntheses and further experiments.

### RT-qPCR analyses of gene expression

We converted approximately 400 ng of RNA per sample into single-stranded cDNA using a mix of oligo-dT20 primers with a high-capacity cDNA reverse transcription kit (Applied Biosystems, Foster city, CA, USA). Two microlitres of cDNA was used as the template for quantitative real-time polymerase chain reaction (qRT-PCR) to determine the transcript levels of *FLC*, *FT*, *ORE1*, *NAP*, *NAC016*, *SGR1*, *NYC1*, *SEN4*, and *SAG12*. The primers are listed in Supplementary Table S1. Real-time PCR was performed in optical 384-well plates using a QuantStudio 6 & 7 flex real-time PCR system with the power SYBR green PCR master mix (Applied Biosystems) over 40 cycles with Tm = 60 °C. Reactions were performed using an initial denaturation step at 95 °C for 10 min, followed by 40 cycles of 15 s at 95 °C and 1 min at 60 °C. Dissociation curve analysis was performed for all of the primer pairs, and all experimental samples yielded a single sharp peak at the amplicon’s melting temperature. Each sample represented two biological replicates, and each of which included two technical replicates. The results were analysed using QuantStudio real-time PCR software v1.0 (Applied Biosystems) with the standard curve method. The relative gene expression levels were normalised against the transcript levels of *Actin2*. Data were expressed according to the normalised expression.

### Availability of data

The data from phenological measurements, meteorology during the experiments, and gene expression analyses used are listed in Supplementary Data S1–S3, respectively.

## Electronic supplementary material

Supplementary Information

Data S1, Data S2, Data S3, Data S4
