# Supplementary material for: Synchronisation of Arabidopsis flowering time and whole-plant senescence in seasonal environments
Source: Sci Rep. 2018 Jul 6;8:10282. doi: 10.1038/s41598-018-28580-x (PMC6035182; doi:10.1038/s41598-018-28580-x)
Supplement: Supplementary file 1 — Supplementary Information [file 41598_2018_28580_MOESM1_ESM.pdf]

Supplementary Information for

"Synchronisation of *Arabidopsis* flowering time and whole-plant senescence in seasonal environments"

by Matin Miryeganeh, Masaki Yamaguchi, and Hiroshi Kudoh

This file contains Supplementary Table S1, Supplementary Fig. S1, Supplementary Fig. S2.

Supplementary Table S1. List of primers used in this study

Primer Name	Primer sequence
ACT2_F	GCGACCAGACAGAGAAAGAAGG
ACT2_R	GATGGAGAAAAGCGGAAGAAGA
FLC_F	CATCATGTGGGAGCAGAAGCT
FLC_R	CGGAAGATTGTCGGAGATTTG
FT_F	AGTATATGGTCGAGAGAGGTCT
FT_R	ACCCTTAGATCGATTGATCTTGT
ORE1_F	AATGAAGCTGTTGCTTGACG
ORE1_R	AGAAATTCCAAACGCAATCC
NAP_F	AGCCATTCACAGCGGTTCA
NAP_R	GCTTACTTGCTCCTCTCTTCTTG
NAC016_F	ATTCACTTCACAGTCAACAGGTG
NAC016_R	GCTGATGAGAACTGGCTCCT
SGR1_F	TGGGCAAATAGGCTATACCG
SGR1_R	CCACCGCTTATGTGACAATG
NYC1_F	GTTAACAGACGCGATGGAGA
NYC1_R	GCCTGGAAAAGAGCTAGGTG
SEN4_F	CTCTCGGTGTCTTGTTTCCA
SEN4_R	GAAGCCATGAATGGAGCTTT
SAG12_F	CAGCTGCGGATGTTGTTG
SAG12_R	CCACTTTCTCCCCATTTTG

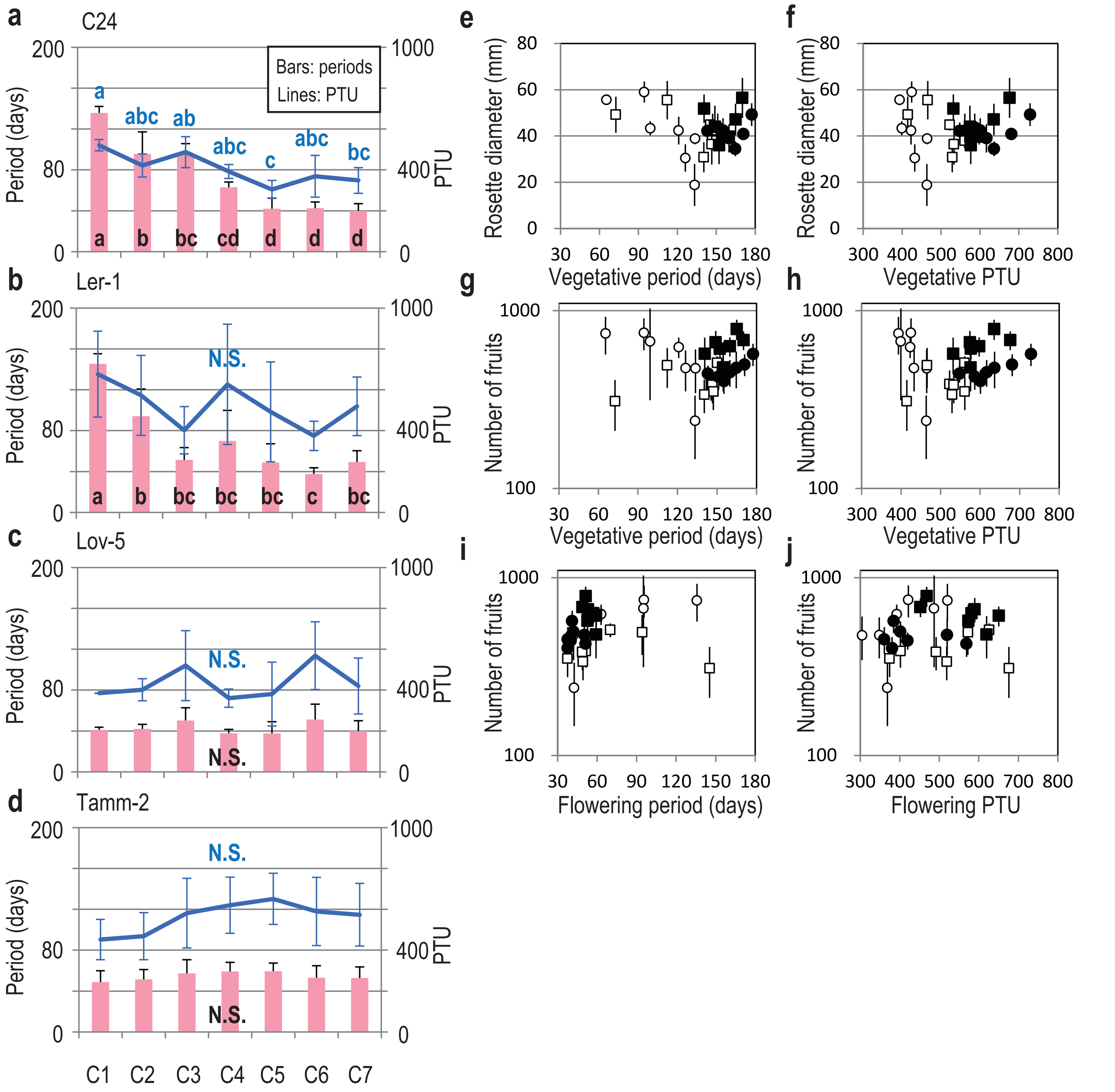

Supplementary Fig. S1| The duration of the flowering periods (red bars) and PTU values during the corresponding periods (lines) are presented for seven cohorts of (**a**) C24, (**b**) Ler-1, (**c**) Lov-5, and (**d**) Tamm-2. (**e, f**) The dependency of the rosette diameter at bolting on the vegetative period and PTU, (**g, h**) dependency of fruit production on the vegetative period and PTU and (**i, j**) on the flowering period and PTU. The durations of the flowering periods were calculated as the number of days from flowering initiation to termination. In **a - d**, the means and standard deviations (SD) are presented. Different letters at the bottom of the bars and next to the lines indicate significant differences ($P < 0.05$) in the periods and PTU between cohorts. N. S. represents no significant difference detected between all combinations of cohorts. In **e - j**, the cohort means are plotted with different symbols for four accessions. The standard deviations of the rosette diameter and number of fruits are represented by vertical bars.

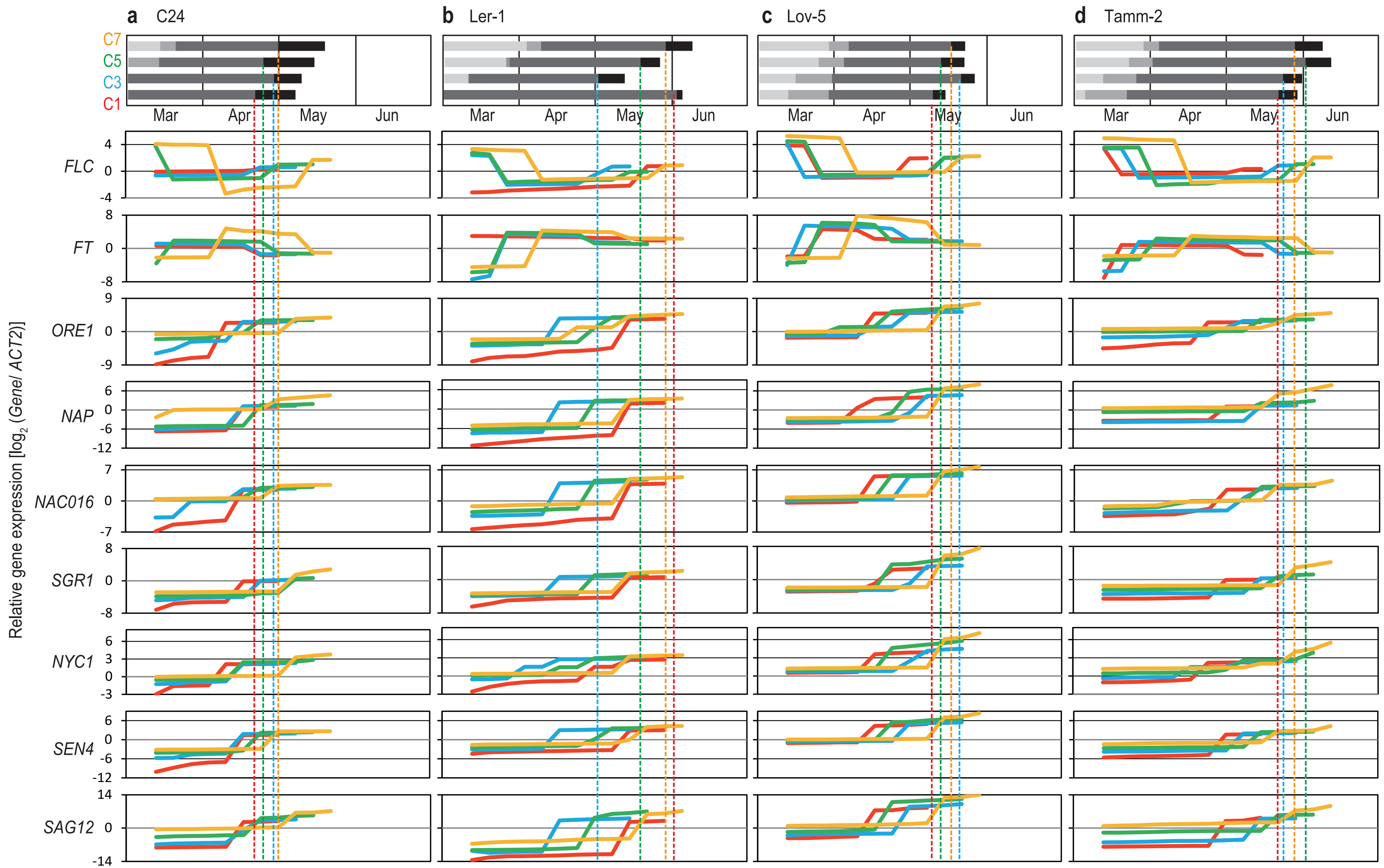

Supplementary Fig. S2| Time-series changes in gene expression of two flowering-time genes (*FLC* and *FT*) and seven senescence-related genes (*ORE1*, *NAP*, *NAC016*, *SGR1*, *NYC1*, *SEN4*, and *SAG12*) before flowering termination for (**a**) C24, (**b**) Ler-1, (**c**) Lov-5, and (**d**) Tamm-2. Gene expression (log2 relative to those of *ACT2*) of the least-senesced leaves of plants was measured weekly from early March. The median values of two replicates at each sampling day are indicated. The average timings of flowering terminations are presented as vertical dashed lines with the corresponding colours. The top diagrams represent the timing of bolting, flowering initiation, flowering termination, and whole-plant senescence (boundaries between light-grey – grey – dark-grey – black, and end of the black bars, respectively) during the period from March–June in the SSE.
